# Language, Paleoneurology, and the Fronto-Parietal System

**DOI:** 10.3389/fnhum.2017.00349

**Published:** 2017-06-30

**Authors:** Emiliano Bruner

**Affiliations:** Programa de Paleobiología, Centro Nacional de Investigación sobre la Evolución HumanaBurgos, Spain

**Keywords:** frontal lobes, parietal lobes, embodiment, visuospatial integration, functional craniology

## Paleoneurology and the frontal lobes

Broca's area has represented a major issue in evolutionary anthropology since the discovery of its association with language impairment. It was generally presumed that the whole frontal lobes had undergone important changes in our phylogenetic lineage, also because of their involvement in personality and executive functions. Accordingly, plenty of authors have declared so far that the fossil record supplies patent evidence of frontal lobe evolution, despite the fact that the fossil record, to date, has supplied none. In terms of frontal sulcal pattern, all human species display a similar scheme, at least from two million years (Tobias, 1987; Holloway, 1995). In terms of volume, there are still disagreements on whether or not humans and living apes share a similar allometric proportion of frontal cortex, and whether any minor difference may be statistically or functionally significant, (e.g., Semendeferi et al., 1997; Rilling, 2006; Barton and Venditti, 2013; Smaers, 2013; Gabi et al., 2016). If there are such critical uncertainties when dealing with living species, it can be easily imagined that these same issue can be particularly difficult to investigate in fossils, which can only provide information on the external gross anatomy of the brain and according to extremely reduced sample sizes. Many statements concerning the evolution of specific frontal cortical traits in fossil hominids are based on individual and fragmented cranial remains. Such punctual and partial information may be useful to delineate further hypotheses, but we don't have to forget that it can only provide incomplete and speculative perspectives (Bruner, 2013). These limitations may generate blurred frontiers between *opinions* (i.e., personal and subjective assessments) and *hypotheses* (perspectives that can be evaluated through experimental or quantitative approaches).

**Figure 1:**
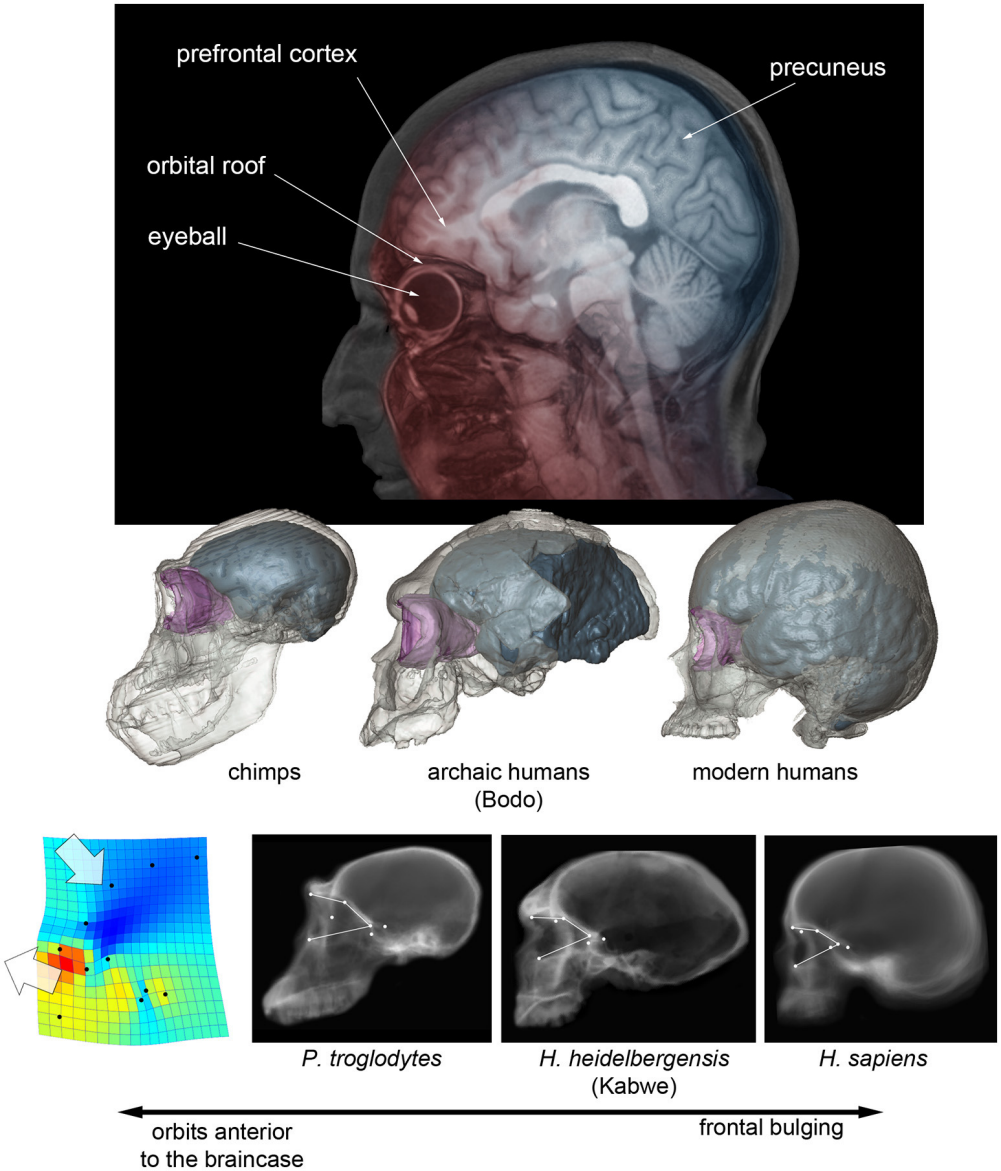
**(Above)** Projected parasagittal MRI slices (in red) showing the spatial relationship between the frontal cortex and upper face in modern humans, and projected midsagittal MRI slices (in blue) showing the parietal, occipital, and cerebellar areas. **(Middle)** Digital reconstruction of a chimp, of an African Middle Pleistocene fossil human, and of a modern human skull, showing the endocranial cavity (blue) and the orbital space (pink) (after Beaudet and Bruner, 2017). **(Below)** CT scout views of chimpanzees, *Homo heidelbergensis* and *Homo sapiens*, showing the position of the orbital boundaries; on the left, the thin-plate spline deformation pattern that separates chimps from modern humans, with fossil humans displaying an intermediate morphology (after Pereira-Pedro et al., 2017).

An actual increase of the frontal or prefrontal cortex volume cannot be tested in fossils because of the many operational limits (like for example the localization of reliable boundaries). Apart from variation in absolute size, Neandertals and modern humans display relatively wider frontal lobes, when compared with other human species (Bruner and Holloway, 2010). In these two species, the breadth of the anterior fossa at the Broca's cap is larger, relative to the general brain width. Therefore, the term “wider” refers to endocranial proportions, and not necessarily to an absolute enlargement or expansion of the lobes. It is worth noting that modern humans and Neandertals are also the only hominids in which the frontal lobes lie entirely above the orbits (Bruner et al., 2014a). The eyeball and the prefrontal cortex are separated by a tiny bony layer (this was the unfortunate principle of lobotomy), and these two districts exert reciprocal spatial constraints during morphogenesis. Orbits are anterior to the braincase in chimps, inferior to the frontal lobes in Neandertals and modern humans, and in an intermediate position in archaic humans (Bruner et al., 2014a; Beaudet and Bruner, 2017-Figure 1). Therefore, we cannot exclude that the lateral frontal widening displayed in modern humans and Neandertals could be a secondary structural consequence (lateral redistribution of the neural mass) of this vertical spatial limitation, with no functional meaning in terms of neural organization. Furthermore, in modern humans, the facial block (the bones forming the face) is much reduced when compared with earlier hominids or apes, and the temporal muscle is reduced accordingly (Cachel, 1978). The Broca's cap is adjacent to the temporal fossa, and the reduction of the muscle further decreases any possible lateral spatial constraints, if any. This does not mean that frontal widening in modern humans and Neandertals was not associated with true brain changes, but only that the influence of cranial architecture cannot be ruled out, and such frontal widening cannot be hence indisputably interpreted as evidence of change in brain organization.

The only specimen which goes against this structural hypothesis is the skull of Maba, found in China and dated to the transition between the Middle and Upper Pleistocene, that shows a facial morphology affine to Neandertals (including anterior fossa which overlap with the orbits), but with a plesiomorph braincase and narrow frontal lobes (Wu and Bruner, 2016). In this case, despite the vertical constraints, the frontal cortex is not wider, possibly supporting the functional (neural adaptation) and not the structural (mass redistribution) hypothesis. However, intra-specific variation for these proportions is noticeable, and larger samples are needed to test hypotheses according to a proper statistical framework. In general, specimens do not prove or reject hypotheses, samples do.

Modern humans also display more bulging frontal squama when compared with other human species, a character which is nonetheless very variable, and with differences that are less pronounced when considering the endocranial profile (Bookstein et al., 1999; Bruner et al., 2013). Also in this case, the increase in frontal curvature is apparently proportional to the displacement of the upper face below the anterior cranial fossa, with fossil humans displaying a phenotype which is intermediate between modern humans and apes (Beaudet and Bruner, 2017; Pereira-Pedro et al., 2017- see Figure 1). The frontal bone integrates the spatial relationship between face and braincase, and it is therefore likely that, also for this character, we are dealing with structural consequences of the cranial architecture and not with a real change in the organization of the frontal lobe.

Also frontal asymmetry, another hallmark of language, cannot help in this sense. All human species in the last 2 million years show a similar asymmetry pattern, in which the frontal lobe is larger on the right side and the occipital lobe is larger on the left side (Holloway, 1980, 1981; Grimaud-Hervé, 1997). This pattern is also found in living apes, although to a minor degree of expression and frequency (Holloway and De La Coste-Lareymondie, 1982). Currently, we cannot exclude the possibility that this increased expression in humans is a secondary consequence of larger brain and allometric effects (Gómez-Robles et al., 2013). It is worth noting that, even in modern humans, the relationship between Broca's area, brain morphology, and cortical asymmetries, is rather blurred and inconsistent (Keller et al., 2009; Amunts and Zilles, 2012). Hence, it is not surprising that the evaluation of this same relationship is even less reliable when dealing with few incomplete skulls belonging to extinct species.

Therefore, apparently there is still no firm evidence of crucial or noticeable morphological changes of the frontal lobes in the human lineage. Of course, the fact that fossils do not reveal any patent variation in these areas does not mean that the frontal lobes have not undergone evolutionary changes in the genus *Homo*. Human-specific traits like the proportions of the prefrontal cortex, the proportions of white matter, extrinsic neural connections, and specific microstructural variations (Schoenemann et al., 2005; Rilling et al., 2012; Passingham et al., 2017) are largely silent to the fossil record, and they cannot be directly evaluated in paleoneurology.

## Parietal lobes and visuospatial integration

In terms of geometry, the most outstanding brain difference among hominids concerns the parietal surface. Modern humans have a larger parietal bone (Bruner et al., 2011) and lager parietal lobes (Bruner et al., 2003; Bruner, 2004). Also Neandertals display wider upper parietal areas, when compared with more archaic human taxa, but not as expanded as in modern humans. In fossils, a detailed analysis of the parietal parcellation is not feasible. Nonetheless, spatial variations seem to deal with the dorsal areas, pointing to the two main folds of the parietal lobe, namely the intraparietal sulcus and the precuneus. The size of the precuneus is extremely variable among adult modern humans, representing a main source of midsagittal morphological diversity due to increase/decrease of its longitudinal extension, which depends on its cortical surface area (Bruner et al., 2014b, 2015, 2017a). This same feature also represents the main midsagittal brain difference between humans and chimps, being much larger in our species (Bruner et al., 2017b). This variation spatially matches the longitudinal bulging observed in the evolution of *H. sapiens* endocranial form. In contrast, the lateral widening of the dorsal parietal lobules, observed in both modern humans and Neandertals, can be tentatively associated with the area occupied by the intraparietal sulcus (Pereira-Pedro and Bruner, 2016). The precuneus is a main hub of brain connectivity, and has a crucial role in bridging somatosensory experience (body) with visual information (environment), integrating mental imagery with self-centered processes in space and time, and even at social level (Cavanna and Trimble, 2006; Margulies et al., 2009; Land, 2014; Peer et al., 2015). The intraparietal sulcus, a fold which is larger and more diversified in humans than apes, is particularly involved in eye-hand coordination and attention (Grefkes and Fink, 2005; Tunik et al., 2007). Most of these parietal functions are generally labeled as *visuospatial integration*, underlying cognitive processes which can be partially investigated in fossils (Bruner and Iriki, 2016; Bruner et al., 2016). Spatial coordination is relevant in language evolution because of a recognized association between speech and manual dexterity (see Binkofski and Buccino, 2004). This perspective has been further emphasized by stressing the importance of shared processes between body experience and language processing (Jirak et al., 2010). According to this view, sensorimotor simulations may link body experience, mirror neurons, and language coding, associating language to “embodied” circuits (Buccino et al., 2005; Marino et al., 2012). Taking into account the possible relevance of body experience in language processing, we should evaluate to what extent language capacity was triggered, facilitated, or improved, by visuospatial capacities. In this case, we should consider whether the fact that Neandertals and modern humans display enlarged visuospatial cortical regions may represent evidence of such association.

## Conclusions

In 1983, Ralph Holloway, in a large and detailed review, explained why paleoneurology and the fossil record cannot give any solution to the debate concerning the evolution of language (Holloway, 1983). He remarked that fossils can supply corroborations, but not proofs, because of the scanty evidence, incomplete information, and lack of quantitative replicable methodologies. Despite the Holloway's frank conclusion, many authors and textbooks have continued to state the opposite. The mantra on the evidence of frontal evolution in human fossils is so rooted in popular feeling that generally the statement is given for granted, and not associated with any accompanying reference. But the relationship between endocranial gross morphology and cognitive processes is partial and imprecise, and fossils can only supply additional integrative support to a more complete scenario, which must be designed according to multiple and independent sources of information. Modern humans and Neandertals both display relatively wider, but probably not relatively larger, frontal lobes. We don't know whether this morphological variation is associated with any functional change. However, both species also displayed changes at the parietal cortex, which was much more apparent and noticeable in modern humans. Changes in the parietal areas may supply additional information on language when recognizing the importance of embodiment and body experience in language coding. As Holloway suggested, changes in the parietal areas may imply changes in social structure which, in humans, is something intimately associated with language. In general, brain size itself may be a good proxy to estimate social and cognitive parameters in primates, also when dealing with language issues (Aiello and Dunbar, 1993). After all, space, time, and social structure are all integrated within shared egocentric (self-centered) schemes based on self-recognition, body relationships, and visuospatial perspectives (Hills et al., 2015; Maister et al., 2015; Peer et al., 2015; Erle and Topolinski, 2017). It must be taken into account that parietal cortex is not only influenced by genetic components (Chen et al., 2012), but it is also particularly sensitive to environmental factors including training and culture, in which ecological, neural, and cognitive elements exert reciprocal and integrated effects (Iriki and Taoka, 2012).

The fronto-parietal system is a complex cerebral network largely based on reciprocal signaling (Caminiti et al., 2015) and with a crucial role in cognitive complexity (Jung and Haier, 2007). Fronto-parietal spatial changes may also influence the general organization of the brain, including connectivity relationships with subcortical areas involved in linguistic capacities (Boeckx and Benítez-Burraco, 2014). It is therefore interesting that both modern humans and Neandertals, the two human species with more derived cultural traits, display species-specific morphological features in both frontal and parietal brain areas.

## Author contributions

The author confirms being the sole contributor of this work and approved it for publication.

### Conflict of interest statement

The author declares that the research was conducted in the absence of any commercial or financial relationships that could be construed as a potential conflict of interest.
